# Marital Status and Loneliness During the Pandemic: The Compensatory Role of Online Social Activities

**DOI:** 10.1093/geroni/igaf122.3116

**Published:** 2025-12-31

**Authors:** Ji Hyun Lee, Jeehoon Kim

**Affiliations:** Montana State University, Bozeman, Montana, United States; Idaho State University, Pocatello, Idaho, United States

## Abstract

An increase in loneliness has been a public concern during the COVID-19 pandemic and heightened loneliness was reported among at-risk groups, including unmarried older adults. When social distancing policies changed the amount and modality of how people connected with others, it is important to examine how online social activities helped alleviate loneliness. The current study examined the effects of online social activities on loneliness for married and unmarried older adults in 2021. Data comes from Round 11 of the National Health and Aging Trends Study (n = 2,849, Age M(SD)=78.4, 55% female, 82 % White, 47% married/partnered). Participants reported their loneliness during the last month with one item (dichotomized as never/less than once a week and more than several days a week). Survey-weighted logistic regression models were performed stratified by marital status. Covariates included age, gender, education, race, income, self-rated health and memory, mental health (PHQ-4), physical activity, close social ties, and various social participation. Unmarried older adults were lonelier (35% vs. married 11%) and less likely to be online. When examining the effects of engagement in three online social activities (email/text, SNS, and video chat) during the last month on loneliness, we found that using video chat with family and friends was associated with lower loneliness among unmarried older adults (OR = 0.65, p =.008). The findings suggest that real-time video-based communication played a compensatory role in reducing loneliness for unmarried older adults. We discuss the value of social technology and its public health implications to reduce the digital divide.

